# Effect of short-term food restriction on iron metabolism, relative well-being and depression symptoms in healthy women

**DOI:** 10.1007/s40519-013-0091-2

**Published:** 2013-12-19

**Authors:** Rafal W. Wojciak

**Affiliations:** 1 Department of Human Nutrition and Hygiene, Poznan University of Life Sciences, Poznan, Poland; 2 Department of Clinical Psychology, Poznan University of Medical Sciences, 70. Bukowska Str., 60-854 Poznan, Poland

**Keywords:** Food restrictions, Women, Iron, Anemia, Mood, Depression

## Abstract

**Aim:**

The idea that iron deficiency anemia can be recognized in depressive patients has been around for a few years, as well as negative association between ferritin levels and depression. Iron deficiency anemia, associated with low iron intake, has been observed in women using restriction diets, for example in vegetarians or anorexics. There are no data on the influence of the short-term food restrictions, observed for example in slimming women, on iron management and its connection with behavior expressed via changes in the subject’s emotional state.

**Materials and methods:**

This study describes the effect of one- and two-day food restrictions (every 8 days for a period of 48 days) on selected iron management parameters in the serum and blood of 46 healthy volunteer women (23 in each group), aged 25.5 ± 3.0 years, in association with the subjects’ self-described emotional status and depression symptoms. The association between iron parameters and depression was also analyzed.

**Results:**

Results show that short-term (2 days) fasting significantly decreases iron concentrations in serum and hair, as well as levels of ferritin, hemoglobin, hematocrit, red blood cells, and total iron binding capacity, but the short-term fasting did not influence the other iron management parameters. Each model of food restrictions also increased negative feelings towards depression. A significant negative correlation between serum ferritin levels and depression was found in women who starved for 2 days.

**Conclusions:**

The study shows that, through an impact on mineral levels, even short-term food restrictions, as observed in many slimming women and girls, can be a reason for iron deficiency and also can alter the emotional status of healthy women. Maybe depression symptoms in anorexia or other eating disorders patients can be associated with iron deficiencies.

## Introduction

In recent times, many people have begun to look for simple methods to regulate their body mass, as part of the fight against overweight and obesity. In our previous studies [1, 2], we observed that more than 80 % of young women who tried different methods of weight loss, such as alternative diets or intensified physical activity, used them at least once a year. Almost half of the women in those studies applied free-will starvation as a method of weight loss for periods ranging from a few days a year to one or even two days a week. Articles about the positive influence of short-term fasting and starvation on the human condition have recently become widespread in popular magazines, yet scientific papers rarely describe this phenomenon. On the other hand, micronutrient deficiencies during fasting were observed in different populations, as a result of an unbalanced diet [3, 4]. Zinc and iron are those trace elements whose levels react very rapidly to their insufficient intake. Iron deficiency anemia can be observed in many undernourished populations, especially in women who are slimming, have vegetarian diets or are pregnant [3, 5, 6]. The relationship of iron to brain function, cognition, and behavior, including affective behavior, has been a subject of interest during the past decades. Iron deficiency anemia is associated with disturbances in behavior related to responsiveness, unhappiness and alertness, especially in infants [7–9]. More recently, disturbances in iron metabolism have been suggested as potential pathological markers in depressed patients [10, 11]. The associations between low serum and blood iron management parameters on the one hand, and symptoms of depression and low mood on the other, are found in slimming obese patients, postpartum patients and malnourished patients as well as those subjects tested after short-term food deprivation [4, 12, 13]. The association between serum ferritin concentrations and depression is still controversial. Some authors [6] have not found such significant associations but others have concluded that depression is associated with decreased levels of serum ferritin and other iron management parameters [14, 15]. On the other hand, many women using starvation as a method of weight loss or to cleanse their bodies are of the opinion that fasting improves their mood and positively influences their quality of life [16]. This is in opposition to the opinion of some authors who have observed the negative influence of food deprivation and malnutrition on mood, depression and other psychological states such as cognitive functions [9, 10, 17, 18].

To address the gap in the literature, this study was conducted to examine the association between short-term food deprivation and the iron status, depression and self-assessed mood of young healthy women.

## Materials and methods

### Ethics

Participants were informed of all procedures of the research, and their consent to participate in the study was confirmed in writing, according to the guidelines of the Bioethical Committee. All procedures were approved by the Bioethical Committee of Poznan University of Medical Sciences, Poznan (No. 16/05).

### Subjects and materials

46 volunteers, healthy women, without symptoms of depression or any previous other psychiatric episodes (assessed by family doctors and psychotherapist), aged 24.5 ± 3.0 years (20–30), were included in the study. The body mass (61.1 ± 4.4, 51.0–78.0 kg), body mass index (BMI) (21.2 ± 1.9, 18.7–23.9 kg/m^2^) and body fat content (electric bio-impedance method—24.5 ± 2.1 %) of the women were measured before and after the study. The body mass, BMI and body fat content after the study in the one-day starvation group were: 59.1 ± 4.0 kg, 20.5 ± 1.5 kg/m^2^, 24.6 ± 2.6 %, respectively, and in two-day starvation group: 58.2 ± 4.3 kg, 19.8 ± 2.1 kg/m^2^, 23.4 ± 2.5 %, respectively. A vast majority of the women (90 %) ate 3–4 meals a day and 10 % had 5–6 meals. The women were randomly divided into two groups (*n* = 23, each), and asked to starve 1 or 2 days for every 8 days of the 48 days of experiment (6 × 1 day of starvation and 6 × 2 days of starvation). All testing procedures were initiated before and after the starvation period. During the days of starvation, none of the participants could eat more than 200 kcal, and were allowed to drink only water with average concentrations of minerals (the approved product and drink lists and their calorie values were given to all subjects, mostly fruits). Minerals, vitamins or other dietary supplements were prohibited. Occipital scalp hair samples (ca. 0.5 g) were taken from all the women in the study and stored in dry conditions until the analytical procedure. The blood from all the subjects was always drawn at 8 a.m., the serum was then separated and stored (in heparinized tubes) as blood samples at −78 °C for <1 month before assay.

### Assessment of relative well-being, mood, and depression

The assessment of the subjects’ relative well-being was carried out using the individual psychological interview method through the use of a metric scale (length of 15 cm). On the selected days, participants marked their current well-being from 0 (completely bad feelings) to the 15 cm (excellent, the best we can imagine) on a line. The results are presented as the mean distance from the extreme-left end of the line expressed in centimeters.

Relative mood feeling was based on the participants’ self-assessment via a questionnaire. On the selected days, the women were asked to respond to the items using a 5-point Likert item (0—strongly disagree, 1—disagree, 2—neither agree nor disagree, 3—agree, 4—strongly agree). The items used in the study were as follows: 1. Items describing general mood—*I am full of energy*, *I feel tired, I need a help, I am in full health and feel strong*; 2. Items describing the relationship with family: *I feel introverted, I prefer to isolate myself, my family supports me strongly, I can always rely on friends*; and 3. Items describing basic emotions: *I feel sad, I feel happy, I feel nervous, I feel calm*.

The results are presented as the mean of the subjects’ answers.

The assessments of relative well-being and mood were carried out on six randomly selected normal eating days and compared with the assessments which were always done on the day after starvation periods. The items in these scales were not directly associated with food cravings but with general emotional status.

All of the participants were also asked to complete a depression scale assessment (BDI—Beck Depression Inventory) [19] 2 days before and after the study. The interpretations of the BDI are as follows: 0–11, no depression; 12–26, mild depression; 27–49, moderate depression; 50–63, heavy depression.

### Iron intake

The participants recorded the nutritional values of the all food and drink consumed for every day of the experiment period. Dietary iron intake was calculated based on the Tables of the Food Product Nutritional Values [20] using the *Dietetic* computer program (v. 2006) and were compared with Estimated Average Requirements (EAR) and Recommended Dietary Allowances (RDA) [21].

### Iron assay

The hair samples were washed according to International Atomic Energy Agency recommendations presented previously [3, 4] and were dried to a dry mass. The hair samples were mineralized in 5 mL HNO_3_ (supra pure, 65 %, w/w, Merck, Germany). After mineralization, the samples were quantitatively transferred to PP vials, using deionized water to give approximately 25 mL. The serum samples were defrosted before analyses. After adequate dilution in deionized water, the iron concentrations in the samples were determined via atomic spectrometry using a graphite furnace spectrometer (AAS-5, Zeiss, Jena, Germany). As control for the iron measurement, a control material was used (*Human Serum*, Randox, UK).

The reference values of the iron content in the hair and serum samples were established on the basis of the published data and laboratory norms [3, 4, 22]: serum-Fe, 50–175 μg/dL; hair-Fe, 10–20 μg/g d.m.; red blood cells (RBC), 4.2–5.4 × 10^12^/L; hemoglobin (Hb), 12–16 g/dL; hematocrit (Hct), 37–47 %; mean corpuscular volume (MCV), 81–99 fL; mean corpuscular hemoglobin (MCH), 27–31 pg; mean corpuscular hemoglobin concentration (MCHC), 33–37 g/dL; total iron binding capacity (TIBC), 306–429 μg/dL; serum ferritin concentration, 1.2–15.0 μg/dL.

### Iron management parameters assay

Blood hematologic analysis (Hb, Hct, RBC, MCV, MCH, and MCHC) was carried out using a CELL-DYN 1700 (Abbott, Illinois, US), and the concentrations of TIBC in the serum were determined by colorimetric method using an Olympus analyzer (Life and Material Science, Europa, GmbH). These parameters were analyzed by a certified, commercial laboratory “*Diagnostyka*”. Serum ferritin concentrations were measured by the sandwich-ELISA method using the standard kits for this protein (Human Ferritin ELISA Kit, Phoenix Pharmaceuticals, USA).

### Data analysis and statistics

*Statistica* (v. 10) and *Microsoft Office Excel* (v. 2007) were used to analyze the data. Data were expressed as the arithmetic mean ± SD. Group differences were assessed using the Student’s *t* test and a Wilcoxon test. A Spearman correlation analysis was employed to examine the relationships between depression and serum ferritin and other iron management parameters. In order to determine the extent of the relationship between the two variables of depression and iron parameter levels, logistic regression was used. Data were deemed significant when *p* < 0.05.

## Results

**Fig. 1:**
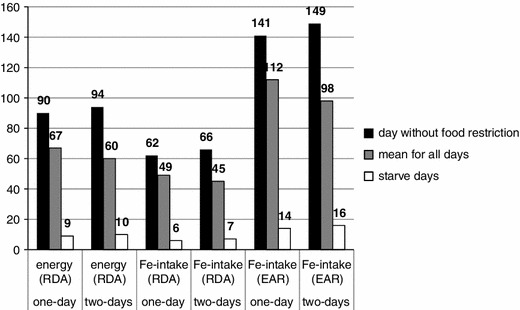
The energy and iron intake as a percentage of the recommendation (%) [20, 21]

The mean intake of energy and iron is presented in Fig. 1. In both groups of women (starved 1 and 2 days for a 8-day period), the mean energy intake of the whole period of the experiment was similar (67 % of RDA), although the women who starved for 2 days consumed slightly more energy than the one-day starvation group on normal diet days. Iron intake, calculated as the mean from all the experiment days, was 50 % of RDA and above 100 % of EAR in both experimental groups. The mean iron intake in days without food restrictions was higher than the mean intake in the whole experiment period. For one-day starvation women, mean iron intake was 62 % of RDA and 141 % of EAR, and for two-day starvation women, the levels were 66 % of RDA and 149 % of EAR.

Table 1 presents the analytical results of iron management parameters. All of these results, measured in both groups of women before and after the study period, lay in the range of normal or reference values. There was no statistically significant influence of one-day starvation on these results in the subjects. The mean concentrations of iron in hair and serum, and ferritin levels did not change after the starvation period, compared to the beginning of experiment (ca. 23 μg/g d.m., 130 mg/dL and 3.8 μg/dL, respectively), nor did the mean blood concentrations of the other iron-dependent parameters: Hb, Hct, RBC, MCV, MCH, MCHC and TIBC (ca. 13 g/dL, 40 %, 4.5 × 10^12^/L, 90 fL, 29, 33 g/dL and 360 μg/dL, respectively). A different situation was observed, however, in the women who starved for 2 days every 8-day period of the 48 days experiment. There were no statistically significant differences between the beginning and the end of the experiment in mean blood concentrations of MCV, MCH, MCHC (ca. 91 fL, 29, 33 g/dL, respectively), while other iron-dependent parameters were significantly lower after the study than before. The mean iron concentrations in hair decreased by approximately 35 % (*p* < 0.001), in serum 15 % (*p* < 0.01), the mean serum ferritin level dropped by approximately 28 % (*p* < 0.001) and the mean blood levels of Hb, Hct, RBC and TIBC all decreased by approximately 8 % (*p* < 0.05), 5 % (*p* < 0.05), 10 % (*p* < 0.05) and 22 % (*p* < 0.01), respectively.

**Table 1:** The concentrations of selected iron management parameters in participants (arithmetic mean ± SD)

Lp.	Parameter	One-day starvation		Two-day starvation	
		Before	After	Before	After
1.	Hair-Fe (μg/g d.m.)	22.5 ± 4.8	23.5 ± 6.2	**22.1** ± 5.4^#^	**14.1** ± 2.0^#^
2.	Serum-Fe (mg/dL)	132.1 ± 5.2	130.4 ± 1.9	**132.8** ± 4.8^^^	**111.2** ± 3.1^^^
3.	Hb (g/dL)	13.1 ± 0.8	12.9 ± 0.8	**13.4** ± 0.7*	**12.3** ± 0.8*
4.	Hct (%)	39.8 ± 3.0	40.1 ± 2.5	**41.7** ± 2.0*	**39.6** ± 1.7*
5.	RBC (×10^12^/L)	4.7 ± 0.7	4.6 ± 0.5	**4.7** ± 0.4*	**4.1** ± 0.3*
6.	MCV (fL)	89.3 ± 3.1	90.1 ± 3.5	90.6 ± 3.2	91.0 ± 4.1
7.	MCH (g/dL)	29.1 ± 1.7	29.2 ± 1.6	29.2 ± 1.8	29.7 ± 1.4
8.	MCHC (g/dL)	33.1 ± 1.1	33.5 ± 1.0	33.0 ± 1.6	33.6 ± 1.2
9.	TIBC (μg/dL)	356.0 ± 78.2	361.1 ± 70.8	**360.2** ± 65.5^^^	**281.0** ± 85.1^^^
10.	Ferritin (μg/dL)	3.8 ± 1.6	3.7 ± 1.4	**3.9** ± 1.8^#^	**2.7** ± 1.7^#^

* Statistically significant differences at *p* < 0.05

^^^ Statistically significant differences at *p* < 0.01

^#^ Statistically significant differences at *p* < 0.001

**Fig. 2:**
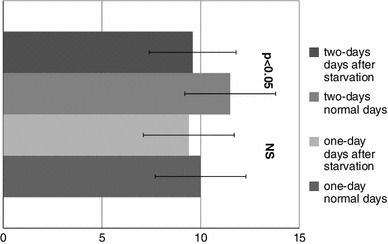
The mean self-assessment of the relative well-being of the subjects in the days with normal eating and directly after starvation (cm)

The assessment of relative well-being, as well as some aspects of mood of the women, is presented in Table 2 and Fig. 2. The analyses of mood and feelings (Table 2) were based on a 4-point scale (from 0 to 4, where “0” disagree and “4” fully agree) and on the questions answered by subjects as important to them. In general, the starvation periods for all subjects, regardless of the number of days of fasting, negatively influenced their feelings and mood. After starving days, the women felt significantly less (*p* < 0.05) healthy, strong and happy and, more tired and nervous, than during normal eating days. These results correspond to the self-assessments of general well-being which were completed by the women after every starvation period (Fig. 2). The women who starved for 2 days felt significantly worse than during the days with normal diet (*p* < 0.05).

**Table 2:** The mean self-assessment of the mood of the subjects on normal eating days and days directly after the starvation

Lp.	Parameter	One-day starvation		Two-day starvation	
		Normal days	Days after starve	Normal days	Days after starve
1.	General mood				
	I am full of energy	**2.8** ± 0.8*	**2.2** ± 0.6*	**3.0** ± 1.0*	**2.3** ± 0.7*
	I feel tired	0.8 ± 0.3	0.9 ± 0.4	**0.7** ± 0.6*	**1.4** ± 0.7*
	I need a help	**0.3** ± 0.4*	**0.9** ± 0.6*	**0.3** ± 0.5*	**0.9** ± 0.5*
	I am in full health and strength	**2.7** ± 1.0*	**2.0** ± 0.5*	**2.9** ± 1.0*	**2.2** ± 1.0*
2.	Relationship				
	I feel introverted	0.2 ± 0.3	0.3 ± 0.4	0.3 ± 0.4	0.5 ± 0.4
	I prefer to isolate	0.2 ± 0.4	0.2 ± 0.4	0.5 ± 0.4	0.7 ± 1.0
	My family supports me	**2.8** ± 1.3*	**2.3** ± 1.3*	2.0 ± 1.1	2.1 ± 1.2
	I can always take on friends	**2.6** ± 1.3*	**2.3** ± 1.3*	1.9 ± 1.0	2.0 ± 1.3
3.	Emotions				
	I feel sad	0.3 ± 0.3	0.5 ± 0.4	0.4 ± 0.4	0.6 ± 0.6
	I feel happy	**2.7** ± 0.7*	**2.0** ± 0.5*	**2.8** ± 0.8*	**2.0** ± 1.0*
	I feel nervous	1.4 ± 1.0	1.3 ± 0.8	**1.5** ± 0.5*	**2.1** ± 0.6*
	I feel calm	2.0 ± 1.0	1.8 ± 0.8	2.3 ± 0.2	2.1 ± 0.9

* Statistically significant differences at *p* < 0.05

**Fig. 3:**
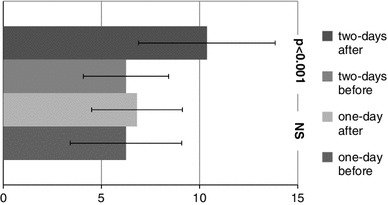
The mean results of depression scale (BDI results)

The mean results of the BDI are presented in Fig. 3. However, the mean results of BDI did not indicate depression in subjects (BDI < 12), in the two-day starvation group, significantly higher scores of depression were observed after the study compared to before (6.25 vs. 10.72, respectively, *p* < 0.01). The women who starved for one day completed the BDI similarly both before and after the study (mean scores ca. 6.50).

**Fig. 4:**
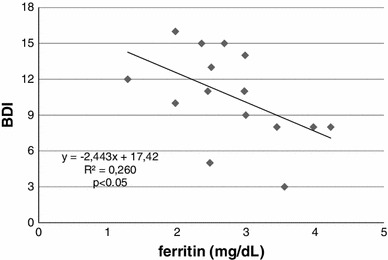
The correlation between ferritin concentration and BDI in the two-day starvation group, measured after the study

Although the correlations between the iron-dependent parameters and depression were looked for in this study, only a statistically significant negative correlation between serum ferritin level and BDI in the women who starved for 2 days was found (Fig. 4, *r* = −0.51, *p* < 0.05).

## Discussion

The study presented here was designed and based on a typical model of starvation used by young women as a simple method of weight loss or “cleaning” the body. Previous studies have researched the most popular types of fasting, and the model of 1 or 2 days of starvation a week has been assessed as the most popular in more than 50 % of young women who were trying to lose weight [1, 2, 16]. However, a lot of popular texts in magazines recommend short-term starvation as a way to decrease food energy, “cleanse” the body and improve well-being. The scientific evidence on this topic is rather inadequate. Previous studies by this author on the distribution of micronutrients in different populations exposed to a reduction in food and energy intake (such as cystic fibrosis patients, hepatitis C virus infected patients treated with interferon, obese children on special low calorie diets, and women with vegetarian diets) have shown significantly lower levels of selected trace elements in hair, serum, and urine compared to the normal or reference values [3, 4, 23, 24]. These observations suggest that in people using free-will starvation as a method of weight loss, similar deficiencies might be observed. Healthy women with normal nutritional habits were asked to participate in this study. Their energy and iron intake was recorded for all experimental days. However, it was observed that energy intake was almost in the recommended range (ca. 90 % of RDA). Furthermore, iron intake was measured at 60 % of RDA and, more than 100 % of EAR. The use of short-term starvation (1 or 2 days) reduced the mean level of energy intake to 70 % of RDA and iron intake to 50 % of RDA and still more than 100 % of EAR. In summary, mean energy and iron consumption were no different between women who starved for 1 or 2 days. This fact suggests that the significant differences in iron management, mood and depression observed in this study were caused by the model of experiment rather than because of iron intake.

Many authors are in the opinion, that common nutritional habits of Polish women are insufficient in the consumption of products such as vegetables, fruits, and dietary fibers [25, 26]. This fact leads to micronutrient deficiencies, especially in trace elements [27–30]. The use of slimming diets by women leads to an insufficient intake of vitamins (thiamin, riboflavin, niacin, ascorbic acid, vitamin A and D) as well as calcium, zinc and iron [28, 31]. Low iron intake can be especially unfavorable for health. Insufficient iron intake is the main cause of iron deficiency anemia in women [6, 29, 32]. Anemia in women with vegetarian diets, pregnant women and women with menstruation problems can be result of iron deficiencies [3, 14, 33]. The iron management results obtained by the study presented above confirm these suggestions. However, although there were no results below the normal range of iron parameters, the two-day starvation period significantly decreased much of them in women in the relatively short period of the experiment (only 48 days, with 12 days of food restriction). The question is: what about women who starve every week? There are, as yet, no data describing this phenomenon.

On the other hand, the effect of short-term food deprivation on a subject’s psychological state has been widely discussed in a number of papers. Stanga et al. [18] have shown the influence of food deprivation on psychological dysfunctions such as depression, anxiety, irritability, apathy, poor sleep patterns, and loss of concentration. Similar observations have been made by other authors working with different patients with periodically or permanently impaired nutrition [12, 17, 34]. Furthermore, Herbert et al. [13] have suggested that even very short-term food deprivation (24 h) intensifies interoceptive awareness, not just restricted to food cues, which is probably affected by feelings of hunger.

Many authors have recently focused on the influence of iron deficiency anemia on depression. Steward and Hirani [35] examined almost 2,000 participants over 65 years. They concluded that lower than normal levels of hemoglobin, ferritin, and transferrin were associated with depressive symptoms. Similar results were observed in patients with maternal iron deficiency anemia which can affect postpartum emotions and cognition [9, 36] as well as postpartum depression [14, 15, 37]. The association between depression and iron management parameters has also been investigated by authors, studying the different populations such as female medical students and adults employees [5, 10, 11], as well as in animal studies [7, 8]. Many of these authors have observed similar results to those presented in this paper regarding the association of ferritin concentrations and depression [10, 11, 14, 35].

Finally, the present results may ultimately have some implications for understanding changes in mood and behavior in people using special diets and fasting. The association between iron deficiency anemia and depression has been widely discussed; however, the influence of short-term starvation on the alteration of iron management is still not fully recognized. On the other hand, the observations of behavior and mood changes in people who use short-term food restriction for different purposes are in no doubt. The results of this study can be a first step to designing further experiments to answer the question of whether short-term starvation is good for a person’s health or not. Does fasting negatively influence on quality of life, and in particular, the emotional status of women? Based on the results obtained in this study, the answer to the second question is yes. Free-will starvation decreases the quality of life rather than increases, opposite to the popular opinion presented by some people using restricted diets.
